# Genomic Frontiers in Alzheimer’s Research: A Primer on the Alzheimer’s Disease Sequencing Project (ADSP) and its AI/ML Opportunities

**DOI:** 10.1002/alz.087888

**Published:** 2025-01-03

**Authors:** Li‐San Wang, Yuk Yee Leung, Wan‐Ping Lee, Amanda B Kuzma, Otto Valladares, William S. Bush, Eden R. Martin, Adam C. Naj, Timothy J. Hohman, Gerard D. Schellenberg, Alzheimer’s Disease Sequencing Project

**Affiliations:** ^1^ University of Pennsylvania Perelman School of Medicine, Philadelphia, PA USA; ^2^ Penn Neurodegeneration Genomics Center, Perelman School of Medicine, University of Pennsylvania, Philadelphia, PA USA; ^3^ Cleveland Institute for Computational Biology, Department of Population and Quantitative Health Sciences, Case Western Reserve University, Cleveland, OH USA; ^4^ John P. Hussman Institute for Human Genomics, University of Miami Miller School of Medicine, Miami, FL USA; ^5^ Department of Biostatistics, Epidemiology, and Informatics, University of Pennsylvania Perelman School of Medicine, Philadelphia, PA USA; ^6^ Penn Neurodegeneration Genomics Center, Philadelphia, PA USA; ^7^ Vanderbilt Memory & Alzheimer’s Center, Department of Neurology, Vanderbilt University Medical Center, Nashville, TN USA; ^8^ Penn Neurodegeneration Genomics Center, Department of Pathology and Laboratory Medicine, University of Pennsylvania Perelman School of Medicine, Philadelphia, PA USA

## Abstract

**Background:**

In this introductory talk, we embark on a journey of through the genomic frontiers of Alzheimer’s research via the revolutionary Alzheimer’s Disease Sequencing Project (ADSP).

**Method:**

ADSP integrates together various components that collectively unravel the intricate genetic landscape of Alzheimer’s disease with the ultimate goal of advancing precision medicine for the millions affected globally by this devastating disease. With a goal of sequencing and analyzing up to 150,000 complete genomes and associated clinical and functional data in the next five years, ADSP has amassed an unprecedented wealth of genomic data from diverse populations, providing a comprehensive and holistic understanding of the genetic underpinnings of Alzheimer’s disease.

**Result:**

This presentation serves as a primer, exploring various components of the ADSP and discuss the unprecedented opportunities it presents for Alzheimer’s disease and AI/ML researchers: (1) Diversity Initiative: The ADSP places a paramount emphasis on diversity, ensuring the inclusion of a wide range of populations in its genomic dataset. (2) Phenotype Harmonization: Harmonizing phenotypic data across diverse cohorts is a critical aspect of the ADSP, facilitating meaningful comparisons and analyses. (3) Functional Genomics: Moving beyond genetic variations, the ADSP incorporates functional genomics to discern the biological mechanisms at play. (4) AI/ML Opportunities: the focus of this session, we will start by illuminating the wide range of opportunities the ADSP offers to AI/ML researchers. From predictive modeling to identifying novel biomarkers and therapeutic targets, we will explore the vast landscape of possibilities for data‐driven discoveries that can shape the future of Alzheimer’s research and precision medicine.

**Conclusion:**

As we uncover the potential of AI/ML within the ADSP, we will emphasize the need for collaborative initiatives between AI/ML researchers and the broader Alzheimer’s research community. The synergy between these fields holds the key to unlocking breakthroughs that can translate genomic insights into tangible clinical advancements.

